# The Effects of Modified Constraint-Induced Movement Therapy in Acute Subcortical Cerebral Infarction

**DOI:** 10.3389/fnhum.2017.00265

**Published:** 2017-05-18

**Authors:** Changshen Yu, Wanjun Wang, Yue Zhang, Yizhao Wang, Weijia Hou, Shoufeng Liu, Chunlin Gao, Chen Wang, Lidong Mo, Jialing Wu

**Affiliations:** ^1^Department of Neurorehabilitation, Department of Neurology, Tianjin Huanhu Hospital, Tianjin Key Laboratory of Cerebrovascular and Neurodegenerative DiseasesTianjin, China; ^2^Department of Rehabilitation Medicine, Tianjin Huanhu Hospital, Tianjin Key Laboratory of Cerebrovascular and Neurodegenerative DiseasesTianjin, China; ^3^Neurological Disease Biobank, Tianjin Neurosurgical Institute, Tianjin Huanhu Hospital, Tianjin Key Laboratory of Cerebrovascular and Neurodegenerative DiseasesTianjin, China

**Keywords:** constraint-induced movement therapy, rehabilitation, motor evoked potentials, cortical reorganization, acute subcortical stroke

## Abstract

**Background**: Constraint-induced movement therapy (CIMT) promotes upper extremity recovery post stroke, however, it is difficult to implement clinically due to its high resource demand and safety of the restraint. Therefore, we propose that modified CIMT (mCIMT) be used to treat individuals with acute subcortical infarction.

**Objective**: To evaluate the therapeutic effects of mCIMT in patients with acute subcortical infarction, and investigate the possible mechanisms underlying the effect.

**Methods**: The role of mCIMT was investigated in 26 individuals experiencing subcortical infarction in the preceding 14 days. Patients were randomly assigned to either mCIMT or standard therapy. mCIMT group was treated daily for 3 h over 10 consecutive working days, using a mitt on the unaffected arm for up to 30% of waking hours. The control group was treated with an equal dose of occupational therapy and physical therapy. During the 3-month follow-up, the motor functions of the affected limb were assessed by the Wolf Motor Function Test (WMFT) and Motor Activity Log (MAL). Altered cortical excitability was assessed via transcranial magnetic stimulation (TMS).

**Results**: Treatment significantly improved the movement in the mCIMT group compared with the control group. The mean WMF score was significantly higher in the mCIMT group compared with the control group. Further, the appearance of motor-evoked potentials (MEPs) were significantly higher in the mCIMT group compared with the baseline data. A significant change in ipsilesional silent period (SP) occurred in the mCIMT group compared with the control group. However, we found no difference between two groups in motor function or electrophysiological parameters after 3 months of follow-up.

**Conclusions**: mCIMT resulted in significant functional changes in timed movement immediately following treatment in patients with acute subcortical infarction. Further, early mCIMT improved ipsilesional cortical excitability. However, no long-term effects were seen.

## Introduction

Stroke significantly increases the mortality and morbidity in the developed as well as developing world (Sudlow and Warlow, 1997; Terént, 2003; Truelsen et al., 2003; Mehndiratta et al., 2015). Despite varying levels of functional recovery, substantial sensorimotor and cognitive deficits persist in more than 50% of survivors, resulting in significant socioeconomic burden (Hendricks et al., 2002; Kim, 2014). Approximately 80% of stroke survivors manifest motor impairments associated with the upper limb (Langhorne et al., 2009; Momosaki et al., 2016). The degree of upper limb paresis is correlated with the basic activities of daily living (ADL) after stroke (Veerbeek et al., 2011; van Mierlo et al., 2016).

Constraint-induced movement therapy (CIMT) promotes movement of upper extremities affected by paralytic stroke. The major components of CIMT include intense repetitive (task-oriented) training and behavioral sharping of the impaired limb with immobilization of the unimpaired arm. Animal studies suggest that increased use of the affected limb overcame the reduced motor activity associated with cortical lesions (Nudo et al., 1996; Kleim et al., 1998). Evidence supports the effectiveness of CIMT in improving dexterity and motor function in individuals with chronic hemiplegia (Wolf et al., 1989; van der Lee et al., 1999; Taub, 2000). There are some limitations to widespread use of CIMT in stroke rehabilitation: first, original CIMT protocol requires constant supervision, therefore, it is more expensive than customary care. Second, original CIMT protocol need constraint of the unaffected hand for approximately 90% of waking hours, but some individuals with hemiplegia cannot tolerate this long limit, and there are also some security issues, especially in acute stroke patients. Compared with original CIMT protocol, the modified CIMT (mCIMT) protocols were feasible and well tolerated in acute stroke patients (Souza et al., 2015).

Although stroke damage can be devastating, many patients survive the initial event and undergo some spontaneous recovery, which can be further augmented by rehabilitative therapy. The first few weeks after stroke are vital for neuroplasticity and relearning of impaired activities (Dobkin, 2004; Kwakkel et al., 2006). Randomized controlled studies have demonstrated mCIMT could improve more affected limb use and function in acute or sub-acute cerebrovascular accident (Page et al., 2005; Singh and Pradhan, 2013).

The recovery of motor function in cortical injury varies from that of subcortical injury (Liu et al., 2015) and the effects of mCIMT in early subcortical ischemic stroke is not established. This study is undertaken to determine if mCIMT is effective in the early phase of subcortical ischemic stroke, and investigate the possible mechanisms underlying the effect. We hypothesized that mCIMT could improve functional outcomes of hemiplegic upper limb in patients with acute subcortical ischemic stroke compared with conventional occupational and physical therapy, and increase ipsilateral cortical excitability.

## Materials and Methods

### Study Setting and Trial Registration

In this single-center randomized controlled clinical trial, patients were recruited from November 2013 to January 2016. This study was approved by the ethics committee of Tianjin Huanhu Hospital. All the procedures involving human participants were approved by the ethics committee of Tianjin Huanhu Hospital and were in accordance with the 1964 Helsinki Declaration and its later amendments, or comparable ethical standards. All participants provided informed consent. In this single-center randomized clinical trial, we compared the upper extremity function between the group exposed to mCIMT and a dose-equivalent control group immediately after intervention and 3 months later. Transcranial magnetic stimulation (TMS) was used to assess changes in cortical excitability after treatment and follow-up. The study was registered with the Chinese Clinical Trial Registry (Registration number: ChiCTR-IOR-15005770).

### Design and Participants

The inclusion criteria were: (1) stroke within 2 weeks of onset; (2) MRI showing subcortical ischemic stroke; (3) ability to raise two fingers with the forearm pronated on the table or lift the wrist 10° or more starting from a fully bent position; (4) respond to a 2-step command; and (5) a Mini Mental State Examination score exceeding 20. The exclusion criteria were: (1) inability to provide informed consent; (2) a history of stroke; (3) deviation greater than 2 cm on the line bisection test; (4) morbidity of the affected upper extremity resulting in functional limitation prior to stroke; (5) life expectancy less than 1 year; or (6) other neurological conditions affecting motor function or assessment (Thrane et al., 2015). Following informed consent, the patients were assigned to mCIMT or the control group using random odd- and even-numbered tickets in sealed envelopes. Patients selected one of the 60 sealed envelopes. Patients who selected tickets with even numbers represented the control group while those with odd numbers were allocated to mCIMT.

### Interventions

The hemiplegic upper extremities in the mCIMT group were trained for 10 days by a licensed occupational therapist. All participants underwent 3 h per day of adaptive task practice and task training of the paretic limb (Wolf et al., 1989; van der Lee et al., 1999; Taub, 2000). Behavioral therapy comprised basic ADL together with skilled functional activities under supervision to improve motor performance. Positive feedback and increased gradations of difficulty were provided. Error data were provided after task training. Tasks with increasing levels of difficulty were assigned. In addition, patients carried a constraining mitt on the unaffected arm for nearly a third of their waking hours.

The control group was exposed to equal doses of traditional occupational therapy and physical therapy using a combination of neurodevelopmental techniques: bimanual tasks for the upper limbs, compensatory techniques for ADLs, strength and range of motion, positioning and mobility training.

### Outcome Measurements

Primary outcomes included upper extremity motor function (tested with Wolf Motor Function Test (WMFT)) and a structured interview of real-world arm use with Motor Activity Log (MAL). The WMFT comprises 15 timed and two strength tasks (lifting the weighted limb and grip strength). The maximum time to complete a task was 120 s. If a trial was incomplete, the result was recorded as 121 s. The median time of all 15 tasks was used for analysis (Morris et al., 2001). The validity and reliability of the test had been demonstrated in stroke populations (Wolf et al., 2001, 2005; Nijland et al., 2010). The MAL was a structured interview comprising 30 standardized questions encompassing various ADL, which was used to assess the subjects’ subjective report of 30 common daily tasks. It included two assessment subscales that rate the more affected upper extremity: an amount of use (AOU) scale and a quality of movement (QOM) scale (Bonifer et al., 2005). The MAL was characterized by stability over a 2-week period with high internal consistency, high inter-rater and test-retest reliability (Taub, 2000). The tool was used extensively in CIMT studies. All participants were assessed after inclusion but before randomization, after 2 weeks and after 3 months.

**Figure 1:**
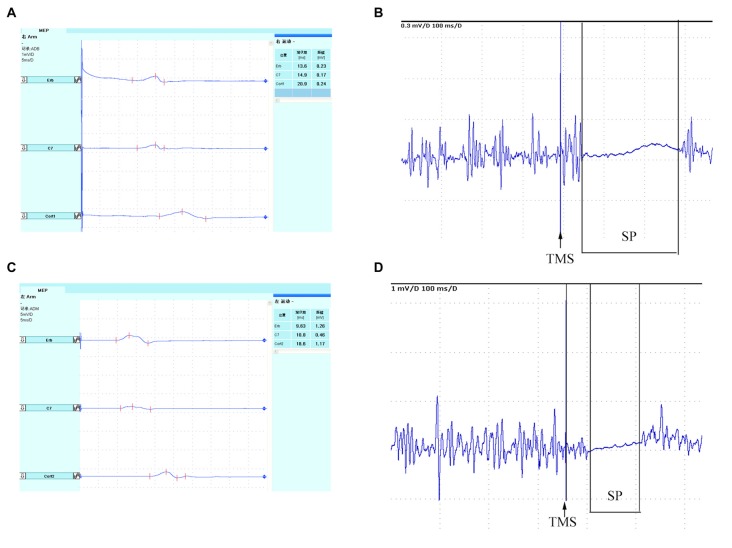
**Schematic diagram of TMS-induced MEP**. Bilateral MEPs and SP recorded in abductor pollicis brevis. Ipsilesional cortical **(A)** MEP and **(B)** SP, and Contralesional cortical **(C)** MEP and **(D)** SP. TMS, transcranial magnetic stimulation; MEP, motor-evoked potential; SP, silent period.

Secondary outcome was the change of cortical excitability. Motor-evoked potentials (MEPs) and cortical silent period (SP) were examined by Dantec Keypoint 4c eletromyography (EMG; Medtronic A/S, Skovlunde, Denmark) with Danish Medtronic MagPro R30 (Medtronic A/S, Skovlunde, Denmark) magnetic stmulator and a focal figure-eight-shaped coil (outer diameter 4.5 cm). The maximum intensity of the magnetic field was 2.5 tesla. All patients were seated comfortably in the supine position. Surface EMG electrodes (filter bandpass: 20–10 kHz) were attached 3 cm apart over the muscle bellies of the abductor pollicis brevis. The 10–20 International electrode system was used for positioning of the TMS coil which located the electrodes on the scalp using standard cranial landmarks. Five stimulation positions were C4/C3, FC4/FC3, C5/C6, CP4/CP3 and C2/C1 according to the 10–20 International electrode system for measurements of cortical excitability. They were marked with an EEG cap, and stimulated with 90% of maximum stimulator output. The position at which stimuli at slightly suprathreshold intensity consistently yielded maximal MEPs in the contralateral abductor pollicis brevis was defined as “hot spot” (Bergmann et al., 2012). Subsequently, rest motor threshold (RMT) was defined according to the guidelines of the International Federation of Clinical Neurophysiology (IFCN) Committee as the minimum stimulus intensity eliciting MEPs of 0.50 mV in the resting muscle in at least 5 of 10 consecutive trials (Chen et al., 2008). The procedures were performed before the intervention and repeated similarly after the intervention and follow-up. Central motor conduction time (CMCT) was a neurophysiological measure that reflected conduction between the primary motor cortex and spinal cord. CMCT was calculated by subtracting the conduction time from the spinal roots to the muscle from the latency of MEPs evoked magnetically by transcranial cortical stimulation (Heald et al., 1993). In our previous study, cortical SP was an useful tool to predict outcome of acute stroke patients (Zhang et al., 2016). The SP had been proposed as an additional factor to the MEP for predicting motor recovery (van Kuijk et al., 2005). The length of the SP was measured from MEP onset until the return of uninterrupted voluntary EMG activity (Uozumi et al., 1991; Trompetto et al., 2000; Figure 1). When TMS was applied during isometric muscle contraction, cortical SP could be evoked following the MEP, which would be lasting up to 100–300 ms (Braune and Fritz, 1995). The intensity of stimulation was 120% RMT. The altered ipsilesional or contralesional MEPs and SPs, were calculated using a change ratio (Δ) as follows:

$$\text{Δ = }\frac{\text{evaluation results post | treatment or follow | up}}{\text{baseline results}}$$

### Statistical Analysis

SPSS version 21.0 package for Windows was used for all statistical analyses. Categorical variables were reported as proportions and continuous variables were reported as median values (interquartile range) or means ± standard deviations (SD). Baseline demographic variables were tested using independent *t*-test or Chi-square test. Differences between within-group inter-group or within-group analysis were determined by Mann-Whitney *U*-test or one-way analysis of variance (ANOVA) followed by Bonferroni multiple comparisons test. The level of statistical significance was set at *P* = 0.05.

## Results

**Figure 2:**
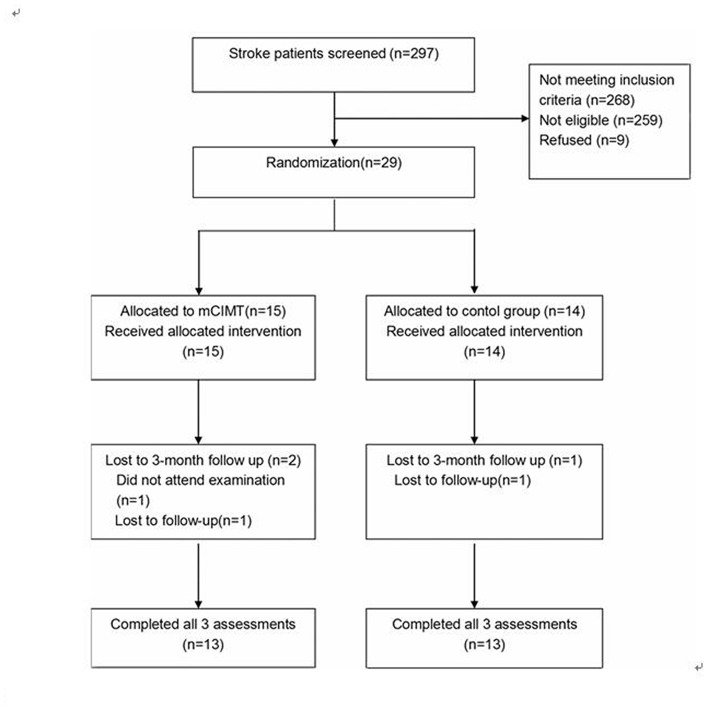
**Flowchart outlining participant selection**.

A total of 297 patients were screened and 29 eligible participants were selected between November 2013 and January 2016. Fifteen patients were assigned to mCIMT and 14 were enrolled in standard therapy. All the participants were inpatients, and no participant dropped out of the post-treatment assessments. One patient refused the 3-month follow-up, and another patient was lost to follow-up in the mCIMT group. Another patient was also lost to follow-up in the standard therapy group after 3 months. The flowchart outlining patient selection is presented in Figure 2.

### Demographic Data

A total of 26 patients (22 men, 4 women) were enrolled and successfully followed up. No significant differences were seen between groups with medical comorbidities: 22 (88.5%) patients with hypertension, 15 (57.7%) with diabetes, three (11.5%) with atrial fibrillation, six (23.1%) with high homocysteine, and seven (27.9%) cases of stenosis of cerebral artery. The disease in the standard therapy group lasted from 2 days to 14 days with a mean of 6.15 ± 3.98 days. In contrast, the CIMT group lasted from 2 days to 14 days with a mean of 7.31 ± 3.86 days. Patient demographic and baseline data are described in Table 1.

**Table 1:** **Baseline characteristics of study participants shown by group**.

	mCIMT	Control group	*P* value
Age (Years)*	58.54 ± 9.61	56.15 ± 11.91	0.579
Sex (M), *n* (%)	11 (84.6)	11 (84.6)	1.000
Smoking, *n* (%)	8 (61.5)	8 (61.5)	1.000
drinking, *n* (%)	7 (53.8)	5 (38.5)	0.431
Hypertension, *n* (%)	11 (84.6)	12 (92.3)	1.000
CardialDiseases, *n* (%)	1 (7.7)	2 (15.4)	1.000
Diabetes, *n* (%)	9 (69.2)	6 (46.2)	0.234
Hyperlipidemia, *n* (%)	19 (63.3)	13 (76.5)	0.353
High homocysteine, *n* (%)	4 (30.8)	2 (15.4)	0.645
Stenosis of cerebral artery, *n* (%)	4 (30.8)	3 (23.1)	0.534
Dominant side affected, *n* (%)	8 (61.5)	7 (53.8)	0.691
NIHSS at admission*	3.85 ± 1.63	3.77 ± 1.59	0.904
Days from stroke onset*	7.31 ± 3.86	6.15 ± 3.98	0.460

*Represents continuous variable with normal distribution, expressed as mean ± SD; other values are expressed as n (%); mCIMT, modified constraint-induced movement therapy; NIHSS, NIH Stroke Scale.

### Clinical Assessment

No significant differences in baseline data (pretreatment) were observed (Table 2). After 2 weeks of intervention, both groups showed an increase in ipsilateral upper limb motor function in the WMFT and MAL compared with baseline. A greater improvement in WMFT scores was observed in the CIMT group than in the standard therapy group (*P* < 0.001), and also in the extent of arm use (*P* = 0.038). However, other items of assessment scales were no different between the two groups. At 3-month follow-up, the scores of the QOM (MAL-QOM) and degree of arm use (MAL-AOU) were no different between mCIMT and standard therapy groups. The WMFT analysis yielded no differences in the functional ability between the groups.

**Table 2:** **Effect of mCIMT on primary and secondary outcomes (*N* = 26)**.

Pre-treatment	Control group	mCIMT group	Value	*p*
WMFT score	2.70 ± 0.87	2.53 ± 1.08	*F* = 0.18	0.674
WMFT time(s)	31.27 ± 18.02	37.23 ± 34.82	*F* = 0.30	0.590
MAL-AOU	0.23 (0.26)	0.27 (0.30)	*U* = −0.28	0.801
MAL-QOM	0.28 (0.34)	0.28 (0.30)	*U* = −0.13	0.897
**Post-treatment**				
WMFT score	3.29 ± 0.90	4.47 ± 0.24	*F* = 20.69	<0.001
WMFT time(s)	16.08 ± 17.50	12.87 ± 14.12	*F* = 0.26	0.611
MAL-AOU	2.39 (1.10)	2.87 (1.72)	*U* = −2.08	0.038
MAL-QOM	1.42 (0.89)	1.76 (0.61)	*U* = −0.90	0.369
**3-month follow-up**				
WMFT score	4.61 ± 0.56	4.71 ± 0.12	*F* = 0.45	0.507
WMFT time(s)	4.38 ± 1.90	3.67 ± 1.44	*F* = 1.19	0.286
MAL-AOM	3.20 (1.18)	3.23 (0.67)	*U* = −0.23	0.840
MAL-QOM	3.27 (0.68)	3.44 (1.20)	*U* = −0.67	0.505

*Normal distribution variable, expressed as mean ± SD; abnormal distribution variable, expressed as median (inter-quartile range). Normal distribution variables were compared by variance analysis. Abnormal distribution variables were compared by Mann-Whitney U-test. mCIMT, modified constraint-induced movement therapy; WMFT time, Wolf Motor Function Test of Performance Time; WMFT score, Wolf Motor Function Test of Functional Ability; MAL-AOU, Motor Activity Log of Amount Of Arm Usage; MAL-QOM, Motor Activity Log of quality of movement*.

### Electrophysiology

TMS revealed similarities between the two groups with respect to baseline data (Table 3). After 2 weeks of intervention, MEPs were present in 10 (76.9%) patients in the CIMT group, with a significant improvement compared with baseline (*P* = 0.047). In the standard therapy group, the MEPs were observed in seven (53.8%) patients, with no difference compared with baseline (*P* = 0.695). Despite the absence of significant differences between the two groups, the presence of MEPs in mCIMT group were significantly higher than the pre-treatment levels. Concurrently, we found that the ipsilesional SP declined 21% compared with the baseline, which was statistically significant compared with the standard therapy group (*P* = 0.029). Other TMS parameters including contralesional SP and CMCT showed no significant changes from the standard therapy group. At 3 months of follow-up, both groups showed significant changes in ipsilesional SP compared with baseline (mCIMT *p* < 0.001; Control group *P* = 0.047). However, no differences were observed compared with each other.

**Table 3:** **Effect of mCIMT on cortical excitability**.

Pre-treatment	Control group	mCIMT group	Value	*p*
MEPs, *n* (%)	5 (38.5)	4 (30.8)		1.000
Ipsilesional SP (ms)	184.7 (164.1)	215.7 (62.6)	*U* = −0.74	0.462
Ipsilesional CMCT (ms)	8.74 ± 1.96	9.25 ± 2.48	*F* = −0.12	0.742
Contralesional SP (ms)	104.4 (121.6)	118.9 (68.8)	*U* = −0.49	0.624
Contralesional CMCT (ms)	8.03 ± 1.47	6.83 ± 2.82	*F* = 1.02	0.347
**Post-treatment**				
MEPs, *n* (%)	7 (53.8)	10 (76.9)		0.411
ΔIpsilesional SP	0.91 ± 0.14	0.69 ± 0.09	*F* = 7.44	0.029
ΔIpsilesional CMCT	0.74 ± 0.29	0.76 ± 0.15	*F* = 0.09	0.929
ΔContralesional SP	1.07 ± 0.12	1.06 ± 0.23	*F* = 0.01	0.948
ΔContralesional CMCT	1.06 ± 0.39	0.77 ± 0.17	*F* = 1.89	0.968
**3-month follow-up**
MEPs, *n* (%)	10 (76.9)	11 (84.6)		1.000
ΔIpsilesional SP	0.74 ± 0.22	0.65 ± 0.08	*F* = 0.56	0.479
ΔIpsilesional CMCT	1.06 ± 0.58	0.89 ± 0.29	*F* = 0.31	0.593
ΔContralesional SP	0.95 ± 0.23	1.03 ± 0.35	*F* = 0.15	0.710
ΔContralesional CMCT	0.96 ± 0.24	0.83 ± 0.28	*F* = 0.65	0.447

*Δ represents altered ratio of TMS motor evoked potentials (MEPs). Normal distribution variable, expressed as mean ± SD; abnormal distribution variable, expressed as median (interquartile range). Normal distribution variables were compared by one way of variance analysis. abnormal distribution variables were compared by Mann-Whitney U-test*.

## Discussion

CIMT and mCIMT are most effective improving functional outcomes of the upper paretic limb (Kwakkel et al., 2015). Intermediate level of evidence supports mCIMT as an effective intervention for upper extremity hemiparesis after stroke (Uswatte et al., 2005). A single clinical trial involving hospitalized patients demonstrated significantly a higher total scores of the Action Research Arm Test and pinch subscale scores in the CIMT group immediately after therapy without follow-up assessment (Dromerick et al., 2000).

In this study, we used two standard clinical tests to assess upper motor function in patients with acute subcortical infarction. We observed a significant increase (of 1.18) in mean WMFT score (*P* < 0.001) in the mCIMT group after intervention (post-treatment) compared with the standard therapy, indicating that mCIMT promoted faster recovery. Although the mean WMFT time was not significantly improved in the mCIMT group after treatment, a downward trend was observed in the mean WMFT time. MAL scores below 0.27 in patients before intervention suggested occasional usage of their more affected arms for ADL tasks. Following intervention, subjects in the mCIMT group showed changes exceeding 2.0 points in the amount of use (AOU-MAL). The results suggested increased use of the affected upper limb for ADL tasks. The AOU scale scores were comparable to the results of previous mCIMT studies (Page et al., 2005; Wu et al., 2007). This study demonstrated that CIMT improved immediate motor function in patients with acute subcortical infarction.

After a follow-up of 3 months, no differences were seen in the WMFT and MAL scores between patients receiving mCIMT and standard therapies. Our result was consistent with a randomized controlled trial, which did not find a favorable effect of CIMT during the 6-month follow-up (Thrane et al., 2015). Recently, a home-based CIMT in patients with upper limb dysfunction after stroke showed that patients in both the groups showed improvement in the QOM. The home CIMT group outscored patients in the standard therapy group at 3 months, which was not consistent with our study. The patients in the home-based CIMT were recruited at least 6 months after stroke, and received 5 h of professional therapy in 4 weeks. Our study recruited patients at an earlier phase of stroke and all patients underwent shorter therapy.

A series of studies demonstrated increased cortical neuroplasticity in the subacute and chronic post-stroke phases of the brain (Liepert et al., 1998; Ro et al., 2006; Boake et al., 2007; Sawaki et al., 2008; Laible et al., 2012). However, studies conducted on patients with subcortical lesions are rare (Jang, 2007). Our study of patients with subcortical infarction displayed improved MEPs and ipsilesional SP in the mCIMT group after treatment, suggesting significant enhancement in cortical excitability of the lesion side. Studies showed that reduced ipsilesional SP level was a prognostic factor for spasticity in chronic stroke (Uozumi et al., 1992; Cruz Martínez et al., 1998). However, it may play a different role in acute stroke. Our earlier study demonstrated decreased ipsilesional SP levels during the first few days after acute cerebral infarction and significantly predicted the outcome within 3 months (Zhang et al., 2016). Patients with an SP value more than 217.05 showed a 7.69-fold increased risk of unfavorable outcomes compared with patients reporting an SP value less than 217.05. In the present study, we found that ipsilesional SP levels were significantly reduced immediately post-mCIMT compared with the control group. We speculate that reduced SP levels may reflect increased muscle tone and improved functional recovery in the acute phase of cerebral infarction.

Previous studies showed that recruitment of supplementary motor areas on the ipsilesional side enhanced the recovery. However, persistent activation of the contralesional cortex was associated with a slower and less complete recovery (Murphy and Corbett, 2009; Xerri, 2012). The present study found no changes in the SP, and CMCT in the contralesional sides of both groups. Our results suggested that the functional improvement of affected upper limb after treatment were associated with enhanced ipsilesional cortical excitability. At 3 months follow-up, significant change in ipsilesional SP was detected in mCIMT group compared with pre-treatment, and we also the similarly change in standard therapy groups. The reason for those changes may be that patients were not required to adhere to similar training at home (Table 4).

**Table 4:** **Within-group changes of electrophysiological parameters**.

	Pre-treatment	Post-treatment	Follow-up	P_1_	P_2_	P_3_
**Control group**						
MEPs, (%)	38.5	53.8	76.9	0.695	0.111	1.000
ΔIpsilesonal SP	1.00	0.92 ± 0.14	0.74 ± 0.22	1.000	0.047	0.244
ΔIpsilesonal CMCT	1.00	0.74 ± 0.29	1.07 ± 0.58	0.892	0.583	1.000
ΔContralesonal SP	1.00	1.07 ± 0.13	0.95 ± 0.23	1.000	1.000	0.732
ΔContralesonal CMCT	1.00	1.06 ± 0.39	0.96 ± 0.24	1.000	1.000	1.000
**mCIMT group**						
MEPs, (%)	30.8	76.9	84.6	0.047	0.015	0.411
ΔIpsilesonal SP	1.00	0.69 ± 0.09	0.65 ± 0.81	<0.001	<0.001	1.000
ΔIpsilesonal CMCT	1.00	0.76 ± 0.15	0.89 ± 0.29	1.000	1.000	1.000
ΔContralesonal SP	1.00	1.05 ± 0.23	1.03 ± 0.35	0.294	1.000	1.000
ΔContralesonal CMCT	1.00	0.77 ± 0.17	0.82 ± 0.26	0.290	0.569	1.000

*Normal distribution variable, expressed as mean ± SD; abnormal distribution variable, expressed as median (interquartile range). Normal distribution variables were compared by variance analysis, and followed by Bonferroni multiple comparisons test. P1 represents statistical difference between Pre-treatment and Post-treatment; P2 represents statistical difference between Pre-treatment and Follow-up; P3 represents statistical difference between Post-treatment and Follow-up*.

Our study limitations are as follows. First, the present study was a single-center clinical trial, and it was difficult to recruit eligible patients. Multi-centered trials are needed to increase the sample size. Second, using non-navigated TMS was a major limitation to the present study. It was well known that navigational systems allow TMS within a spatial deviation of few millimeters to a desired region of the cortex (Rossini and Rossi, 2007; Sparing et al., 2008). Using the International 10–20 electrode system for positioning of the TMS coil was not as accurate as navigational systems, but it was easily applicable in its practicable use. Moreover, using 10–20 electrode system enabled us not only to quickly retrieve the cortical region of interest but also shorten pre-evaluation related time, and avoid some security concerns resulting from navigated TMS in acute stroke patients. Third, we assessed changes of cortical excitability using TMS. However, single TMS pulses were unlikely to distinguish between stimulation of cortico-spinal, intra-cortical and trans-cortical elements, but instead target all three to varying degrees (Bestmann and Krakauer, 2015). We could not determine the contribution of other areas of the brain to the resultant output. Combination of TMS and brain functional imaging or specific stimulation protocols, such as paired-pulse stimulation could facilitate our understanding of neurophysiological mechanisms of stroke recovery.

## Conclusion

Compared with standard therapy, mCIMT induced significant functional changes in acute subcortical ischemic stroke patients. Early intervention with mCIMT promotes ipsilesional cortical reorganization, without any long-term effect.

## Author Contributions

JW contributed to the conception and design of the work; contributed in revising the work for important intellectual content. CY, WW, YZ, YW, WH, SL, CG, CW and LM contributed in the data acquisition. CY, WW and JW contributed in the analysis and interpretation of data for the work. CY and WW contributed in drafting the work. All authors approved the final version to be published, and agree to be accountable for all aspects of the work in ensuring that questions related to the accuracy or integrity of any part of the work are appropriately investigated and resolved.

## Conflict of Interest Statement

The authors declare that the research was conducted in the absence of any commercial or financial relationships that could be construed as a potential conflict of interest.
